# *Bioconductor* toolchain for reproducible bioinformatics pipelines using *Rcwl* and *RcwlPipelines*

**DOI:** 10.1093/bioinformatics/btab208

**Published:** 2021-03-27

**Authors:** Qiang Hu, Alan Hutson, Song Liu, Martin Morgan, Qian Liu

**Affiliations:** Department of Biostatistics & Bioinformatics, Roswell Park Comprehensive Cancer Center, Buffalo, NY 14203, USA; Department of Biostatistics & Bioinformatics, Roswell Park Comprehensive Cancer Center, Buffalo, NY 14203, USA; Department of Biostatistics & Bioinformatics, Roswell Park Comprehensive Cancer Center, Buffalo, NY 14203, USA; Department of Biostatistics & Bioinformatics, Roswell Park Comprehensive Cancer Center, Buffalo, NY 14203, USA; Department of Biostatistics & Bioinformatics, Roswell Park Comprehensive Cancer Center, Buffalo, NY 14203, USA

## Abstract

**Summary:**

The Common Workflow Language (CWL) is used to provide portable and reproducible data analysis workflows across different tools and computing environments. We have developed *Rcwl*, an *R* interface to CWL, to provide easier development, use and maintenance of CWL pipelines from within *R*. We have also collected more than 100 pre-built tools and pipelines in *RcwlPipelines*, ready to be queried and used by researchers in their own analysis. A single-cell RNA sequencing preprocessing pipeline demonstrates use of the software.

**Availability and implementation:**

Project website: https://rcwl.org (Rcwl: https://bioconductor.org/packages/Rcwl; RcwlPipelines: https://bioconductor.org/packages/RcwlPipelines).

**Supplementary information:**

Supplementary data are available at *Bioinformatics* online.

## 1 Introduction

The bioinformatics community increasingly relies on ‘workflow’ frameworks to manage large and complex biomedical data (Di Tommaso et al., 2017; Köster and Rahmann, 2012). One solution facilitating portable, reproducible and scalable workflows across a variety of software and hardware environments is the Common Workflow Language (CWL) (Amstutz et al., 2016). The CWL has been widely adopted by large biomedical projects such as The Cancer Genome Atlas (Weinstein *et al.*, 2013) and Galaxy (Afgan et al., 2018). However, as a domain-specific language, the implementation of CWL requires a level of expertise that is often beyond the capabilities of wet-lab researchers and even skilled data scientists. In addition, the impact of CWL pipelines is weakened by poor integration with downstream statistical analysis tools such as *R* and *Bioconductor* (Amezquita et al., 2020; Huber et al., 2015).

Here, we introduce a *Bioconductor* toolchain for use and development of reproducible bioinformatics pipelines in CWL using *Rcwl* and *RcwlPipelines*. *Rcwl* provides a familiar *R* interface to, and expands the scope of, CWL. *Rcwl* enables best practices and standardized data flow between different tools, and promotes modularization for easy sharing of established pipelines or critical steps. *RcwlPipelines* manages a collection of commonly used bioinformatics tools and pipeline recipes based on *Rcwl*. *RcwlPipelines* develops a community-driven platform for open source, open development and open review of best-practice CWL bioinformatics pipelines. *Rcwl* and *RcwlPipelines* reduces the learning curve required to apply findable, accessible, interoperable and reusable principles to the analysis of multi-omics biological experiments, and to promote community-wide sharing of cloud-ready bioinformatics workflows.

## 2 Implementation

**Fig. 1.**
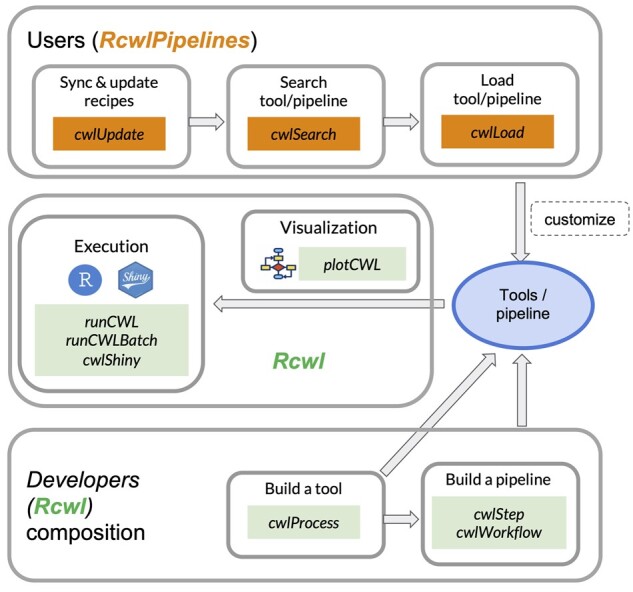
Rcwl & RcwlPipelines functionalities and implementation

*Rcwl* is implemented using the *R* programming language and *Bioconductor* S4 data infrastructure, aiming to facilitate data analysts’ ability to build, read, write and execute diverse computational tools and pipelines using the CWL specification. *Rcwl* defines three major categories of functions: composition, visualization and execution (Fig. 1) to construct CWL pipelines, execute them in various computing environments (e.g. on HPC, cloud or through Shiny application), and visualize the pipeline for reporting purposes. Configuration parameters, docker requirements (da Veiga Leprevost et al., 2017; Grüning *et al.*, 2018) and job schedulers (e.g. SGE, SLURM) are all managed in *R* for enhanced reproducibility and scalability. The tools and pipelines developed by *Rcwl* are readily submitted and executed through *R* as CWL scripts. Beyond just an interface to CWL for wrapping command-line tools, *Rcwl* expands the scope of CWL by enabling direct integration of user-defined *R* functions to use, e.g. established *R/Bioconductor* package functions, as components of the pipeline.

*RcwlPipelines* manages a collection of CWL tools and pipelines developed using *Rcwl* (there are 113 tools and 26 pipelines available at time of writing). *RcwlPipelines* covers a broad range of bioinformatics areas and provides highly customizable pipelines. For example, the neoantigen prediction pipeline integrates tools for DNA sequence alignment, RNA read quantification, variant calling, annotation, phasing and HLA typing. A set of *RcwlPipelines* core functions (Fig. 1) helps users to search and load the best-fit tools for their own data analysis. Expanded examples of code for use of the core functions are provided as Supplementary Material and as *Bioconductor* package vignettes.

## 3 Usage

Development and use involve three steps: (i) (developers only) build the CWL tools by specifying the base command, input/output parameters, and docker requirements. (ii) Connect *Rcwl* tools into pipelines by specifying data flow between steps (using the ‘+’ function). (iii) Execute the pipeline by assigning values to input parameters and specifying the file path to collect the final results. Jobs can be run on single (*runCWL*) or multiple (*runCWLBatch*) computing nodes through the internal integration of *BiocParallel* (Morgan et al., 2020). An extended example is given in the Supplementary Material, where we present a modified version of the pre-processing pipeline of Single-Cell RNA sequencing Data (Tekman et al., 2020) using STARsolo for alignment and quantification, and *DropletUtils* (Lun *et al.*, 2019) for filtering empty droplets from the raw gene-barcode matrix.

## 4 Conclusion

Here, we introduce a *Bioconductor* toolchain for simplified development, usage and maintenance of CWL bioinformatics pipelines using *Rcwl* and *RcwlPipelines*. The packages provide a route to turn any command-line tools into *R* tools as well as integrating existing *R* package functionalities into CWL pipelines, and thus greatly lowered the bar for data analysts to conduct complex bioinformatics workflows directly in *R*.

We are dedicated to implementing more user-oriented core functions and infrastructures, and expanding the current collection of tools and pipelines available to meet wider biomedical research needs. In the future, we will be implementing more specific developing guidelines and benchmark datasets to enhance the reusability and high-quality of pipelines contributed by the community.

## Supplementary Material

btab208_Supplementary_Data
