# Supplementary material for: Bioconductor toolchain for reproducible bioinformatics pipelines using Rcwl and RcwlPipelines
Source: Bioinformatics. 2021 Mar 27;37(19):3351–2. doi: 10.1093/bioinformatics/btab208 (PMC8504628; doi:10.1093/bioinformatics/btab208)
Supplement: btab208_Supplementary_Data [file btab208_supplementary_data.pdf]

Supplementary material: Bioconductor toolchain for reproducible bioinformatics pipelines using Rcwl and RcwlPipelines

Qiang Hu *[*1], ***Alan Hutson*** [1], ***Song Liu*** [1], ***Martin Morgan*** [1], ***and Qian Liu*** [†1]

[1]Roswell Park Comprehensive Cancer Center, Buffalo, NY

[*]`Qiang.Hu@RoswellPark.org` [†]`Qian.Liu@RoswellPark.org`

last compiled: 2021-02-26

Contents

1 Introduction 2

2 scRNA-seq data source 2

3 `RcwlPipelines` core functions 3

3.1 `cwlUpdate` 3

3.2 `cwlSearch` 4

3.3 `cwlLoad`. 4

4 `Rcwl` tool development and S4 class 5

5 scRNA-seq preprocessing tools. 6

5.1 Indexing 6

5.2 Alignment 7

5.3 Count filtering 8

6 scRNA-seq preprocessing pipeline 10

7 Additional functionalities. 12

7.1 Submit parallel jobs 12

7.2 Shiny interface 14

8 Summary. 14

9 SessionInfo 14

1 Introduction

Here we introduce the *Bioconductor* toolchain for usage and development of reproducible bioinformatics pipelines using packages of Rcwl and RcwlPipelines. `Rcwl` provides a simple way to wrap command line tools and build CWL data analysis pipelines programmatically within *R*. It increases the ease of use, development, and maintenance of CWL pipelines. `RcwlPipelines` manages a collection of more than a hundred of pre-built and tested CWL tools and pipelines, which are highly modularized with easy customization to meet different bioinformatics data analysis needs.

As an example, here we demonstrate the usage of a scRNA-seq data preprocessing pipeline (available in `RcwlPipelines`) that uses STARsolo for alignment and quantification, and DropletUtils for filtering raw gene-barcode matrix and removing empty droplets. This pipeline demonstrates the typical use case of our packages. More examples and tutorials can be found on the project website.

2 scRNA-seq data source

The scRNA-seq data source is the 1k PBMCs from 10x genomics. 10x Genomics has its own preprocessing pipeline `Cell Ranger` to process the scRNA-seq outputs it produces to perform the demultiplexing and quantification. However, it requires much configuration to run and is significantly slower than other mappers. In this tutorial, we will use STARsolo to produce a count matrix from FASTQ, and DropletUtils to produce a high-quality count matrix with feature/cell annotation files saved in an *R* object of `SingleCellExperiment`. Before these 2 steps, a one-time indexing step using STARindex is also included.

The dataset used in this tutorial are sub-sampled from the source files to contain only 15 cells instead of 1000. We have also further curated the fastq data to only include reads on chromosome 21, so that the real execution of our Rcwl tools/pipelines in *R* can be completed within 1~2 minutes for each step for demo purposes.

For the mapping, we need a "whitelist" of known cell barcodes. Here we used the 15 barcodes that we already subsetted ("subset15_demo_barcode.txt")

We will be using the hg19 (GRCh37) version of the human genome, and therefore need the hg19 GTF file to annotate our reads (curated on chromosome 21 only for this tutorial).

Data can be loaded from the dedicated GitHub repository.

```
library(git2r)
clone("https://github.com/rworkflow/testdata", "rcwl_data_supp")
```

```
path <- "rcwl_data_supp"  ## source data
dir(path)
## [1] "chr21.fa"
## [2] "Homo_sapiens.GRCh37.75.21.gtf"
## [3] "subset15_chr21_pbmc_1k_v3_S1_L001_R1_001.fastq.gz"
## [4] "subset15_chr21_pbmc_1k_v3_S1_L001_R2_001.fastq.gz"
## [5] "subset15_chr21_pbmc_1k_v3_S1_L002_R1_001.fastq.gz"
## [6] "subset15_chr21_pbmc_1k_v3_S1_L002_R2_001.fastq.gz"
## [7] "subset15_demo_barcode.txt"
```

We create an output directory to save result files from running the tool/pipeline.

```r
outpath <- "outdir"
dir.create(outpath, showWarnings = FALSE)
```

3 RcwlPipelines core functions

For usage of the existing tools/pipelines, three major steps are needed: 1) search and load the tools/pipelines, 2) assign values for each of the defined parameters, 3) execute the tools/pipelines. All steps are done programmatically in *R*, and we can get the results ready in the user-specified directory.

Here we show the usage of 3 core functions: `cwlUpdate`, `cwlSearch` and `cwlLoad` for updating, searching, and loading the needed tools or pipelines in *R*.

3.1 cwlUpdate

The `cwlUpdate` function syncs the current `Rcwl` recipes and returns a `cwlHub` object which contains the most updated `Rcwl` recipes. The `mcols()` function returns all related information about each available tool or pipeline. Currently, we have integrated 113 command line tools and 26 pipelines.

The recipes will be locally cached, so users don't need to call `cwlUpdate` every time unless they want to use a tool/pipeline that is newly added to `RcwlPipelines`. In this example, we are using the Bioc 3.13 which is the current devel version.

```r
library(Rcwl)
library(RcwlPipelines)
atls <- cwlUpdate(branch = "dev", force = TRUE) ## sync the tools/pipelines.
atls
## cwlHub with 139 records
## cache path:  ~/Library/Caches/Rcwl
## # last modified date:  2021-02-22
## # cwlSearch() to query scripts
## # cwlLoad('title') to load the script
## # additional mcols(): rid, rpath, Type, Container, mtime, ...
##
##            title
##   BFC2772 | pl_alignMerge
##   BFC2773 | pl_AnnPhaseVcf
##   BFC2774 | pl_BaseRecal
##   BFC2775 | pl_bwaAlign
##   BFC2776 | pl_bwaMMRecal
##   ...       ...
##   BFC2906 | tl_VarScan2
##   BFC2907 | tl_vcf_expression_annotator
##   BFC2908 | tl_vcf_readcount_annotator
##   BFC2909 | tl_vep
##   BFC2910 | tl_vt_decompose
##          Command
##   BFC2772 bwaAlign+mergeBamDup
##   BFC2773 VCFvep+dVCFcoverage+rVCFcoverage+VCFexpression+PhaseVcf
```

```
##   BFC2774 BaseRecalibrator+ApplyBQSR+samtools_index+samtools_flagstat+samt...
##   BFC2775 bwa+sam2bam+sortBam+idxBam
##   BFC2776 bwaAlign+mergeBamDup+BaseRecal
##   ...     ...
##   BFC2906
##   BFC2907 vcf-expression-annotator
##   BFC2908 vcf-readcount-annotator
##   BFC2909 vep
##   BFC2910 vt decompose
table(mcols(atls)$Type)
##
## pipeline     tool
##       26      113
```

3.2 cwlSearch

We can use (multiple) keywords to search for specific tools/pipelines of interest, which internally search the `mcols()` of “rname”, “rpath”, “fpath”, “Command” and “Containers”.

```
tls <- cwlSearch(c("STAR", "index"))
mcols(tls)
## DataFrame with 2 rows and 14 columns
##           rid        rname         create_time         access_time
##   <character>  <character>         <character>         <character>
## 1     BFC2789 pl_rnaseq_Sf 2021-02-26 20:56:51 2021-02-26 20:56:51
## 2     BFC2891 tl_STARindex 2021-02-26 20:57:06 2021-02-26 20:57:06
##                   rpath       rtype                  fpath last_modified_time
##             <character> <character>            <character>          <numeric>
## 1 /Users/qi31566/Libra..       local /Users/qi31566/Libra..                 NA
## 2 /Users/qi31566/Libra..       local /Users/qi31566/Libra..                 NA
##          etag   expires        Type               Command
##   <character> <numeric> <character>           <character>
## 1          NA        NA    pipeline fastqc+STAR+sortBam+..
## 2          NA        NA        tool                  STAR
##                 Container               mtime
##               <character>         <character>
## 1                      NA 2021-02-22 14:45:16
## 2 quay.io/biocontainer.. 2021-02-22 14:45:16
```

3.3 cwlLoad

The last core function `cwlLoad` loads the `Rcwl` tool/pipeline into the *R* working environment. The code below loads the tool called `STARindex` to index the genome.

```
STARindex <- cwlLoad(title(tls)[2])  ## "tl_STARindex"
STARindex <- cwlLoad(mcols(tls)$fpath[2])  ## equivalent to the above.
```

4 `Rcwl` tool development and S4 class

The *R* object of `STARindex` is a S4 class of `cwlProcess`. It contains the cwl configuration parameters, docker requirements of specific versions, input parameter arguments with pending values upon users' input (some are with default values), and output globbing patterns to be used to pass intermediate files between pipelines steps.

```
STARindex
## class: cwlProcess
##  cwlClass: CommandLineTool
##  cwlVersion: v1.0
##  baseCommand: STAR
## requirements:
## - class: DockerRequirement
##   dockerPull: quay.io/biocontainers/star:2.7.5a--0
## arguments: --runMode genomeGenerate
## inputs:
##   genomeDir (string): --genomeDir STARindex
##   genomeFastaFiles (File): --genomeFastaFiles
##   sjdbGTFfile (File): --sjdbGTFfile
##   runThreadN (int): --runThreadN 4
## outputs:
## outIndex:
##   type: Directory
##   outputBinding:
##     glob: $(inputs.genomeDir)
```

Some utility functions can help to extract specific information.

```
cwlVersion(STARindex)
## [1] "v1.0"
inputs(STARindex)
## inputs:
##   genomeDir (string): --genomeDir STARindex
##   genomeFastaFiles (File): --genomeFastaFiles
##   sjdbGTFfile (File): --sjdbGTFfile
##   runThreadN (int): --runThreadN 4
outputs(STARindex)
## outputs:
## outIndex:
##   type: Directory
##   outputBinding:
##     glob: $(inputs.genomeDir)
requirements(STARindex)
## [[1]]
## [[1]]$class
## [1] "DockerRequirement"
##
## [[1]]$dockerPull
## [1] "quay.io/biocontainers/star:2.7.5a--0"
```

To develop an `Rcwl` tool, developers need to specify the base command (name of command line tools), input parameters (with command prefix, data type, and an id, etc.), and output parameters (with globbing patterns to extract for later steps). The scripts for developing all available tools/pipelines in `RcwlPipelines` are in this GitHub repository including the one for STARindex.

5 scRNA-seq preprocessing tools

5.1 Indexing

Before read alignment and quality control, a one-time genome indexing needs to be done. The command line using `STAR` will look like this:

```
$ STAR --runMode genomeGenerate --runThreadN 4 --genomeDir STARindex
--genomeFastaFiles chr21.fa --sjdbGTFfile Homo_sapiens.GRCh37.75.21.gtf
```

We can equivalently index the genome using the `Rcwl` tool of `STARindex` within *R*, which was internally passed as cwl scripts, by only assigning values to the input parameters, and execute the cwl script using one of the execution functions, e.g., `runCWL` in the local computer. Then the output files are ready to pass as input to the next tool for single cell read alignment.

```
STARindex$genomeFastaFiles <- file.path(path, "chr21.fa")
STARindex$sjdbGTFfile <- file.path(path, "Homo_sapiens.GRCh37.75.21.gtf")
runCWL(cwl = STARindex, outdir = file.path(outpath, "STARindex_output"), docker = TRUE)
## List of length 3
## names(3): command output logs
dir(file.path(outpath, "STARindex_output"), recursive = TRUE)  ## output files
##  [1] "STARindex/chrLength.txt"            "STARindex/chrName.txt"
##  [3] "STARindex/chrNameLength.txt"        "STARindex/chrStart.txt"
##  [5] "STARindex/exonGeTrInfo.tab"         "STARindex/exonInfo.tab"
##  [7] "STARindex/geneInfo.tab"             "STARindex/Genome"
##  [9] "STARindex/genomeParameters.txt"     "STARindex/Log.out"
## [11] "STARindex/SA"                       "STARindex/SAindex"
## [13] "STARindex/sjdbInfo.txt"             "STARindex/sjdbList.fromGTF.out.tab"
## [15] "STARindex/sjdbList.out.tab"         "STARindex/transcriptInfo.tab"
```

Note that the `docker` argument in `runCWL` function takes 4 values: 1) TRUE (default, recommended), which automatically pulls docker images for the required command line tools. 2) FALSE, if users have already pre-installed all required command line tools. 3) "singularity" if the running environment doesn't support docker but singularity. 4) "udocker" for the docker-like runtime without any administrator privileges.

```
STARindex  ## input parameter values (e.g., filepath) are added.
## class: cwlProcess
##  cwlClass: CommandLineTool
##  cwlVersion: v1.0
##  baseCommand: STAR
## requirements:
## - class: DockerRequirement
##   dockerPull: quay.io/biocontainers/star:2.7.5a--0
## arguments: --runMode genomeGenerate
```

```
## inputs:
##   genomeDir (string): --genomeDir STARindex
##   genomeFastaFiles (File): --genomeFastaFiles /Users/qi31566/workspace/rcwl_ms/rcwl_data_supp/chr21.fa
##   sjdbGTFfile (File): --sjdbGTFfile /Users/qi31566/workspace/rcwl_ms/rcwl_data_supp/Homo_sapiens.GRCh37.75
##   runThreadN (int): --runThreadN 4
## outputs:
## outIndex:
##   type: Directory
##   outputBinding:
##     glob: $(inputs.genomeDir)
```

The translation of the `Rcwl` tool into CWL scripts are easily enabled by `writeCWL`, which generates 2 files in the specified directory: 'xx.cwl' for configuration and 'xx.yml' for parameter values. The prefix of the two files takes the name of the input `cwlProcess` object by default, and can be specified using the "prefix" argument.

```
writeCWL(STARindex, prefix = "STARindex", outdir = outpath)
##                   cwlout                  ymlout
## "outdir/STARindex.cwl" "outdir/STARindex.yml"
dir(outpath, pattern = "cwl|yml")
## [1] "STARindex.cwl" "STARindex.yml"
```

5.2 Alignment

Now we can follow the previous example to load our `Rcwl` tool of `STARsolo` to align reads to the indexed genome.

```
cwlSearch("STARsolo")
## cwlHub with 2 records
## cache path:  ~/Library/Caches/Rcwl
## # last modified date:  2021-02-22
## # cwlSearch() to query scripts
## # cwlLoad('title') to load the script
## # additional mcols(): rid, rpath, Type, Container, mtime, ...
##
##              title                   Command
##   BFC2792 | pl_STARsoloDropletUtils STARsolo+DropletUtils
##   BFC2892 | tl_STARsolo             STAR
STARsolo <- cwlLoad("tl_STARsolo")
```

```
cdna.fastq <- file.path(path, list.files(path, pattern = "_R2_"))
cb.fastq <- file.path(path, list.files(path, pattern = "_R1_"))
cblist <- file.path(path, "subset15_demo_barcode.txt")
genomeDir <- file.path(outpath, "STARindex_output/STARindex")

inputs(STARsolo)
## inputs:
##   readFilesIn_cdna (File[]): --readFilesIn
##   readFilesIn_cb (File[]):
##   genomeDir (Directory): --genomeDir
##   whiteList (File): --soloCBwhitelist
```

```
##   soloType (string): --soloType CB_UMI_Simple
##   soloUMIlen (string): --soloUMIlen 12
##   runThreadN (int): --runThreadN 1
STARsolo$readFilesIn_cdna <- cdna.fastq
STARsolo$readFilesIn_cb <- cb.fastq
STARsolo$whiteList <- cblist
STARsolo$genomeDir <- genomeDir

runCWL(STARsolo, outdir = file.path(outpath, "STARsolo_output"))
## List of length 3
## names(3): command output logs
dir(file.path(outpath, "STARsolo_output"), recursive = TRUE)
##  [1] "Aligned.out.sam"                    "Log.final.out"
##  [3] "Log.out"                            "Log.progress.out"
##  [5] "SJ.out.tab"                         "Solo.out/Barcodes.stats"
##  [7] "Solo.out/Gene/Features.stats"       "Solo.out/Gene/filtered/barcodes.tsv"
##  [9] "Solo.out/Gene/filtered/features.tsv" "Solo.out/Gene/filtered/matrix.mtx"
## [11] "Solo.out/Gene/raw/barcodes.tsv"     "Solo.out/Gene/raw/features.tsv"
## [13] "Solo.out/Gene/raw/matrix.mtx"       "Solo.out/Gene/Summary.csv"
## [15] "Solo.out/Gene/UMIperCellSorted.txt"
```

The output files generated in the "STARsolo_output" folder can now be passed into the next tool for QC.

5.3 Count filtering

To get a high-quality count matrix we apply the `DropletUtils` *Bioconductor* package, which will produce a filtered dataset that is more representative of the `Cell Ranger` pipeline.

Since CWL itself doesn't support the integration of *R* packages or *R* function, this is a unique feature for `Rcwl`, where we can easily connect the upstream data preprocessing steps (previously based on command line tools) and the downstream data analysis steps (heavily done in *R/Bioconductor*).

The idea here is to put anything you need into a user-defined *R* function, with specified arguments for input and output files, then it's ready to be wrapped as an `Rcwl` tools for execution.

For example, in wrapping the *Bioconductor* package DropletUtils functionalities, we wrote this `Rcwl` tool called tl_DropletUtils with 3 major steps: 1) use the `read10xCounts` function to read the raw aligned files and convert into a `SingleCellExperiment` object. 2) calculate the barcode ranks and plotting. 3) calculate the empty droplets and plotting. We have also defined the tool output to collect the `SingleCellExperiment` Rdata file and diagnostic figures pdf file.

```
library(DropletUtils)
DropletUtils <- cwlLoad("tl_DropletUtils")
inputs(DropletUtils)
## inputs:
##   dirname (Directory): dir.name=
##   lower (int): lower= 100
##   df (int): df= 20
```

```
DropletUtils$dirname <- file.path(outpath, "STARsolo_output/Solo.out")
DropletUtils$lower <- 100
DropletUtils$df <- 5
runCWL(DropletUtils, outdir = file.path(outpath, "dropletUtils_output"), showLog = FALSE)
## List of length 3
## names(3): command output logs
dir(file.path(outpath, "dropletUtils_output"))
## [1] "diagnostics.pdf"  "sce_filtered.rds"
```

Now that we get 2 output files:

1. The pdf file with 2 diagnostic figures: Barcode ranks, and empty droplets. For details regarding interpretation of each diagnostic figure, please refer to the DropletUtils vignette.

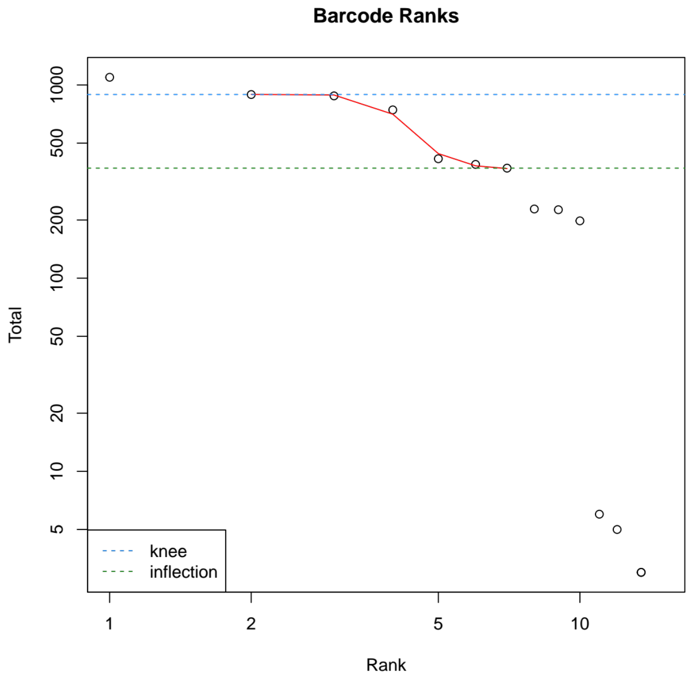

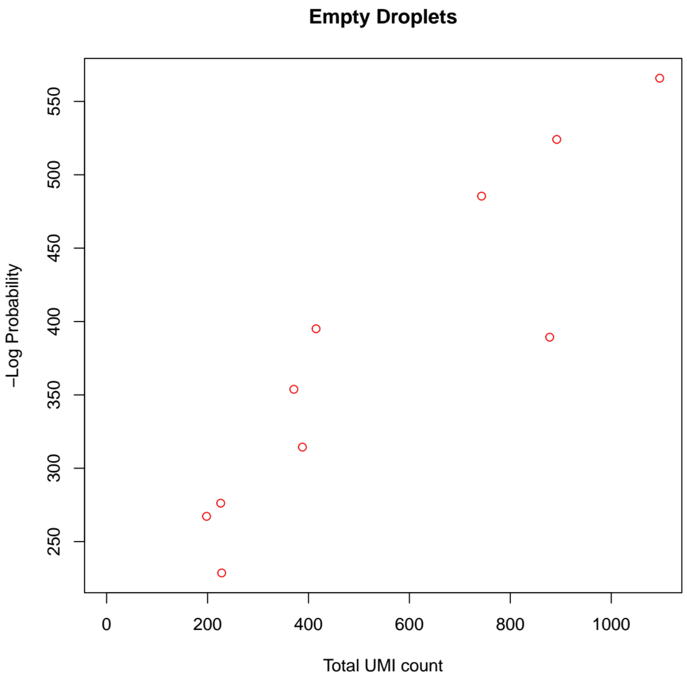

2. The `SingleCellExperiment` object which has filtered out unqualified cells and is analysis ready.

```
sce <- readRDS(file.path(outpath, "dropletUtils_output/sce_filtered.rds"))
sce
## class: SingleCellExperiment
## dim: 736 10
## metadata(1): Samples
## assays(1): counts
## rownames(736): ENSG00000238411 ENSG00000264462 ... ENSG00000230982
##   ENSG00000212932
## rowData names(3): ID Symbol Type
## colnames: NULL
## colData names(2): Sample Barcode
## reducedDimNames(0):
## altExpNames(0):
```

6 scRNA-seq preprocessing pipeline

Alternatively and more easily, we can use the pipeline called `pl_STARsoloDropletUtils` (recipe here) for the scRNA-seq data preprocessing. This pipeline integrated the `STARsolo` and `DropletUtils` for a streamlined preprocessing analysis within *R*. It is included in `RcwlPipelines` and is ready to be customized for your own research. Pipelines can be visualized by the `plotCWL` function.

```
STARsoloDropletUtils <- cwlLoad("pl_STARsoloDropletUtils")
```

```r
plotCWL(STARsoloDropletUtils)
```

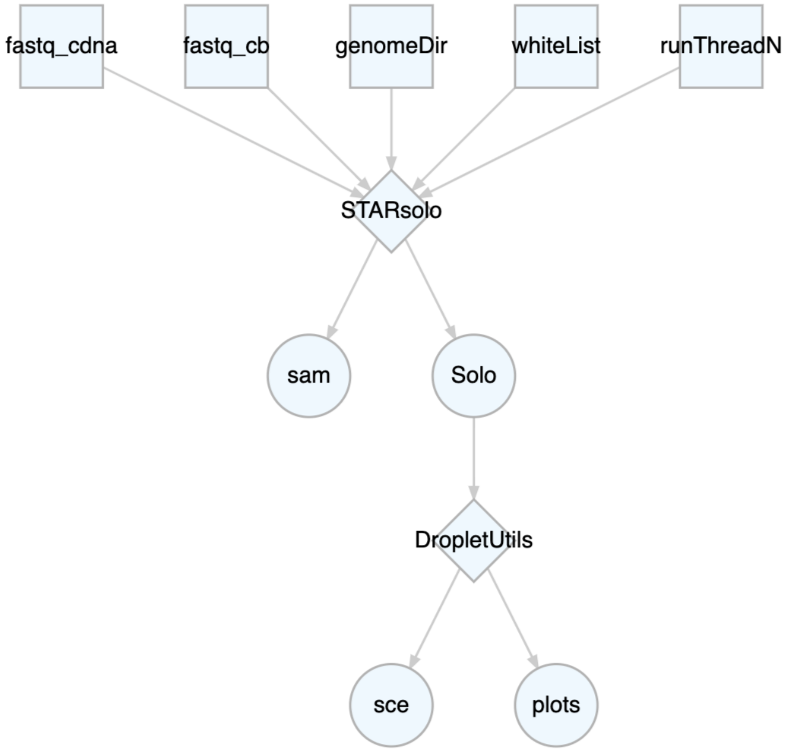

For `Rcwl` pipelines, we only need to assign input values for the whole pipeline, not individual tools involved. The input and output between each step are pre-defined in the pipeline to ensure a smooth passing.

```r
inputs(STARsoloDropletUtils)
## inputs:
##   fastq_cdna (File[]):
##   fastq_cb (File[]):
##   genomeDir (Directory):
##   whiteList (File):
##   runThreadN (int):
STARsoloDropletUtils$fastq_cdna <- cdna.fastq
STARsoloDropletUtils$fastq_cb <- cb.fastq
STARsoloDropletUtils$genomeDir <- file.path(outpath, "STARindex_output/STARindex")
STARsoloDropletUtils$whiteList <- cblist
STARsoloDropletUtils$runThreadN <- 1

runCWL(STARsoloDropletUtils, outdir = file.path(outpath, "scpipeline_output"))
## List of length 3
## names(3): command output logs
```

The overall output of the pipeline was pre-defined to glob the important files from separate steps.

```
outputs(STARsoloDropletUtils)
## outputs:
## sam:
##   type: File
##   outputSource: STARsolo/outAlign
## Solo:
##   type: Directory
##   outputSource: STARsolo/Solo
## sce:
##   type: File
##   outputSource: DropletUtils/outsce
## plots:
##   type: File
##   outputSource: DropletUtils/plots
dir(file.path(outpath, "scpipeline_output"), recursive = TRUE)
##  [1] "Aligned.out.sam"                     "diagnostics.pdf"
##  [3] "sce_filtered.rds"                    "Solo.out/Barcodes.stats"
##  [5] "Solo.out/Gene/Features.stats"        "Solo.out/Gene/filtered/barcodes.tsv"
##  [7] "Solo.out/Gene/filtered/features.tsv" "Solo.out/Gene/filtered/matrix.mtx"
##  [9] "Solo.out/Gene/raw/barcodes.tsv"      "Solo.out/Gene/raw/features.tsv"
## [11] "Solo.out/Gene/raw/matrix.mtx"        "Solo.out/Gene/Summary.csv"
## [13] "Solo.out/Gene/UMIperCellSorted.txt"
```

7 Additional functionalities

7.1 Submit parallel jobs

Powered by `BiocParallel`,`Rcwl` supports parallel job running for multiple samples using the `runCWLBatch` function. The following example demonstrates how to do the parallel alignment for the 2 samples using the `STARsolo` tool.

The `BPPARAM` argument in `runCWLBatch()` defines the parallel parameters. It can be defined by `BiocParallel::BatchtoolsParam` function, where the `cluster` argument takes different values for different cluster job manager, such as "multicore", "sge" and "slurm". More details about available options can be checked by `?BiocParallel::BatchtoolsParam`.

```
library(BiocParallel)
```

```
bpparam <- BatchtoolsParam(workers = 2, cluster = "sge",
                           template = batchtoolsTemplate("sge"))
```

In the following example, we are using "multicore" for the parallel running.

The `inputList` argument is required to be a list of input parameter values for samples that are to be computed parallelly. **NOTE** that the names of the list must be consistent with the ids of input parameters. In this example, the names are `readFilesIn_cdna` and `readFilesIn_cb`.

The `paramList` argument is required to be a list of input parameter values that are to be shared for all parallelly running samples.

```
bpparam <- BatchtoolsParam(workers = 2, cluster = "multicore")
input_lst <- list(readFilesIn_cdna = list(
                      sample1 = cdna.fastq,
                      sample2 = cdna.fastq),
                  readFilesIn_cb = list(
                      sample1 = cb.fastq,
                      sample2 = cb.fastq)
                  )
param_lst <- list(whiteList = cblist,
                  genomeDir = genomeDir,
                  runThreadN = 2)
res <- runCWLBatch(cwl = STARsolo,
                   outdir = file.path(outpath, "STARsolo_batch_output"),
                   inputList = input_lst, paramList = param_lst,
                   BPPARAM = bpparam)
```

The results are saved in separate folders for each parallel sample.

```
dir(file.path(outpath, "STARsolo_batch_output"), recursive = TRUE)  ## output files
##  [1] "sample1/Aligned.out.sam"
##  [2] "sample1/Log.final.out"
##  [3] "sample1/Log.out"
##  [4] "sample1/Log.progress.out"
##  [5] "sample1/SJ.out.tab"
##  [6] "sample1/Solo.out/Barcodes.stats"
##  [7] "sample1/Solo.out/Gene/Features.stats"
##  [8] "sample1/Solo.out/Gene/filtered/barcodes.tsv"
##  [9] "sample1/Solo.out/Gene/filtered/features.tsv"
## [10] "sample1/Solo.out/Gene/filtered/matrix.mtx"
## [11] "sample1/Solo.out/Gene/raw/barcodes.tsv"
## [12] "sample1/Solo.out/Gene/raw/features.tsv"
## [13] "sample1/Solo.out/Gene/raw/matrix.mtx"
## [14] "sample1/Solo.out/Gene/Summary.csv"
## [15] "sample1/Solo.out/Gene/UMIperCellSorted.txt"
## [16] "sample2/Aligned.out.sam"
## [17] "sample2/Log.final.out"
## [18] "sample2/Log.out"
## [19] "sample2/Log.progress.out"
## [20] "sample2/SJ.out.tab"
## [21] "sample2/Solo.out/Barcodes.stats"
## [22] "sample2/Solo.out/Gene/Features.stats"
## [23] "sample2/Solo.out/Gene/filtered/barcodes.tsv"
## [24] "sample2/Solo.out/Gene/filtered/features.tsv"
## [25] "sample2/Solo.out/Gene/filtered/matrix.mtx"
## [26] "sample2/Solo.out/Gene/raw/barcodes.tsv"
## [27] "sample2/Solo.out/Gene/raw/features.tsv"
## [28] "sample2/Solo.out/Gene/raw/matrix.mtx"
## [29] "sample2/Solo.out/Gene/Summary.csv"
## [30] "sample2/Solo.out/Gene/UMIperCellSorted.txt"
```

7.2 Shiny interface

`cwlShiny()` opens a user-friendly shiny interface for running any `Rcwl` tools or pipelines. By default, users need to put in the absolute file path for each input parameter. **NOTE**, multiple file paths need to be separated by colon. Click the `run` button, it will start running and return the output file paths under `Output` tag. Users can also check the `Command` and `Log` in the shiny interface page.

```
cwlShiny(STARsolo)
```

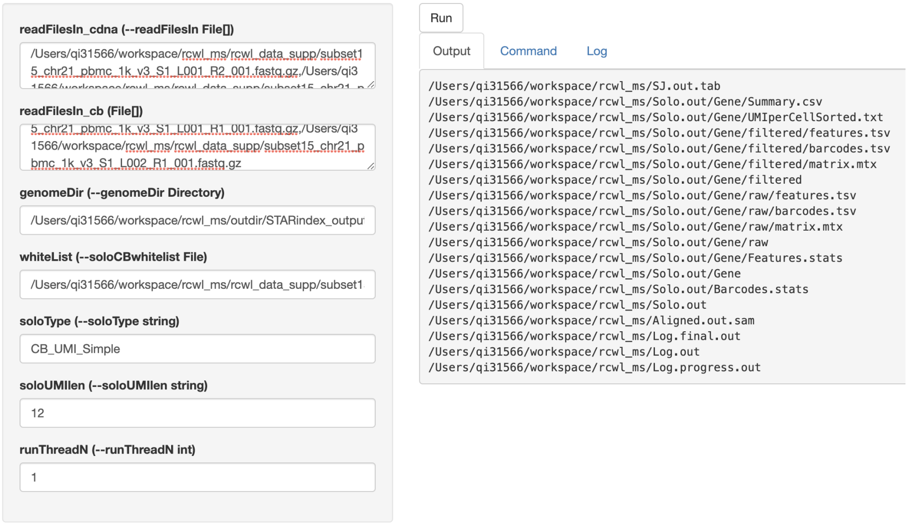

8 Summary

Here we have introduced the usage of two packages: `Rcwl` and `RcwlPipelines`, in constructing and executing the CWL tools/pipelines within *R* for the previously command line tools as well as customized *R* functions. The pre-built tools and pipelines are highly modularized and optimized for easy customization for specific data analysis needs. These packages are under active development, and we welcome any question for the functionalities, feature requests (support site, email), and issue reports.

Importantly, we are trying to make this project as a community effort for developing and sharing of specific sets of tools and pipelines in their bioinformatics domains. We look forward to any collaborations in developing the pipelines and please feel free to make your pull requests for your recipes here.

9 SessionInfo

```
sessionInfo()
## R version 4.0.3 (2020-10-10)
## Platform: x86_64-apple-darwin13.4.0 (64-bit)
## Running under: macOS Catalina 10.15.7
##
```

```
## Matrix products: default
## BLAS/LAPACK: /Users/qi31566/miniconda3/envs/r-base/lib/libopenblasp-r0.3.12.dylib
##
## locale:
## [1] en_US.UTF-8/en_US.UTF-8/en_US.UTF-8/C/en_US.UTF-8/en_US.UTF-8
##
## attached base packages:
## [1] parallel  stats4    stats     graphics  grDevices utils     datasets
## [8] methods   base
##
## other attached packages:
##  [1] DropletUtils_1.10.3         SingleCellExperiment_1.12.0
##  [3] SummarizedExperiment_1.20.0 Biobase_2.50.0
##  [5] GenomicRanges_1.42.0        GenomeInfoDb_1.26.2
##  [7] IRanges_2.24.1              MatrixGenerics_1.2.1
##  [9] matrixStats_0.58.0          BiocStyle_2.18.1
## [11] BiocParallel_1.24.1         RcwlPipelines_1.7.7
## [13] BiocFileCache_1.14.0        dbplyr_2.1.0
## [15] Rcwl_1.7.12                 S4Vectors_0.28.1
## [17] BiocGenerics_0.36.0         yaml_2.2.1
##
## loaded via a namespace (and not attached):
##   [1] ellipsis_0.3.1            rprojroot_2.0.2
##   [3] scuttle_1.0.4             XVector_0.30.0
##   [5] fs_1.5.0                  rstudioapi_0.13
##   [7] remotes_2.2.0             bit64_4.0.5
##   [9] fansi_0.4.2               sparseMatrixStats_1.2.1
##  [11] codetools_0.2-18          R.methodsS3_1.8.1
##  [13] cachem_1.0.4              knitr_1.31
##  [15] pkgload_1.2.0             jsonlite_1.7.2
##  [17] R.oo_1.24.0               HDF5Array_1.18.1
##  [19] shiny_1.6.0               DiagrammeR_1.0.6.1
##  [21] BiocManager_1.30.10       compiler_4.0.3
##  [23] httr_1.4.2                dqrng_0.2.1
##  [25] basilisk_1.2.1            backports_1.2.1
##  [27] assertthat_0.2.1          Matrix_1.3-2
##  [29] fastmap_1.1.0             limma_3.46.0
##  [31] cli_2.3.1                 later_1.1.0.1
##  [33] visNetwork_2.0.9          htmltools_0.5.1.1
##  [35] prettyunits_1.1.1         tools_4.0.3
##  [37] igraph_1.2.6              glue_1.4.2
##  [39] GenomeInfoDbData_1.2.4    dplyr_1.0.4
##  [41] batchtools_0.9.15         rappdirs_0.3.3
##  [43] Rcpp_1.0.6                rhdf5filters_1.2.0
##  [45] vctrs_0.3.6               DelayedMatrixStats_1.12.3
##  [47] xfun_0.21                 stringr_1.4.0
##  [49] ps_1.5.0                  beachmat_2.6.4
##  [51] testthat_3.0.2            mime_0.10
##  [53] lifecycle_1.0.0           devtools_2.3.2
##  [55] edgeR_3.32.1              zlibbioc_1.36.0
##  [57] basilisk.utils_1.2.2      hms_1.0.0
```

```
##  [59] promises_1.2.0.1          rhdf5_2.34.0
##  [61] RColorBrewer_1.1-2        curl_4.3
##  [63] memoise_2.0.0             reticulate_1.18
##  [65] stringi_1.5.3             RSQLite_2.2.3
##  [67] desc_1.2.0                checkmate_2.0.0
##  [69] filelock_1.0.2            pkgbuild_1.2.0
##  [71] rlang_0.4.10              pkgconfig_2.0.3
##  [73] bitops_1.0-6              evaluate_0.14
##  [75] lattice_0.20-41           Rhdf5lib_1.12.1
##  [77] purrr_0.3.4               htmlwidgets_1.5.3
##  [79] bit_4.0.4                 processx_3.4.5
##  [81] tidyselect_1.1.0          magrittr_2.0.1
##  [83] bookdown_0.21             R6_2.5.0
##  [85] generics_0.1.0            base64url_1.4
##  [87] DelayedArray_0.16.1       DBI_1.1.1
##  [89] pillar_1.5.0              withr_2.4.1
##  [91] RCurl_1.98-1.2            tibble_3.0.6
##  [93] crayon_1.4.1              utf8_1.1.4
##  [95] rmarkdown_2.7             progress_1.2.2
##  [97] usethis_2.0.1             locfit_1.5-9.4
##  [99] grid_4.0.3                data.table_1.14.0
## [101] blob_1.2.1                callr_3.5.1
## [103] git2r_0.28.0              digest_0.6.27
## [105] xtable_1.8-4              tidyr_1.1.2
## [107] httpuv_1.5.5              brew_1.0-6
## [109] R.utils_2.10.1            sessioninfo_1.1.1
```
